# RNA-Seq of *Borrelia burgdorferi* in Multiple Phases of Growth Reveals Insights into the Dynamics of Gene Expression, Transcriptome Architecture, and Noncoding RNAs

**DOI:** 10.1371/journal.pone.0164165

**Published:** 2016-10-05

**Authors:** William K. Arnold, Christina R. Savage, Catherine A. Brissette, Janakiram Seshu, Jonathan Livny, Brian Stevenson

**Affiliations:** 1 Department of Microbiology, Immunology and Molecular Genetics, University of Kentucky School of Medicine, Lexington, KY, United States of America; 2 Department of Biomedical Sciences, University of North Dakota School of Medicine and Health Sciences, Grand Forks, ND, United States of America; 3 Department of Biology, University of Texas at San Antonio, San Antonio, TX, United States of America; 4 Broad Institute of MIT and Harvard, Cambridge, MA, United States of America; University of Maryland, College Park, UNITED STATES

## Abstract

*Borrelia burgdorferi*, the agent of Lyme disease, differentially expresses numerous genes and proteins as it cycles between mammalian hosts and tick vectors. Insights on regulatory mechanisms have been provided by earlier studies that examined *B*. *burgdorferi* gene expression patterns during cultivation. However, prior studies examined bacteria at only a single time point of cultivation, providing only a snapshot of what is likely a dynamic transcriptional program driving *B*. *burgdorferi* adaptations to changes during culture growth phases. To address that concern, we performed RNA sequencing (RNA-Seq) analysis of *B*. *burgdorferi* cultures at early-exponential, mid-exponential, and early-stationary phases of growth. We found that expression of nearly 18% of annotated *B*. *burgdorferi* genes changed significantly during culture maturation. Moreover, genome-wide mapping of the *B*. *burgdorferi* transcriptome in different growth phases enabled insight on transcript boundaries, operon structures, and identified numerous putative non-coding RNAs. These RNA-Seq data are discussed and presented as a resource for the community of researchers seeking to better understand *B*. *burgdorferi* biology and pathogenesis.

## Introduction

*Borrelia burgdorferi*, the spirochetal bacterium that causes Lyme disease, persists in nature through a life cycle that necessitates alternating infection of vertebrates and ticks [1]. Successful infection of these two very different types of animals, and transmission between host and vector, requires that *B*. *burgdorferi* express different sets of host-interactive proteins at each step of the cycle. During the past two decades, numerous borrelial proteins have been shown to be expressed specifically during mammalian or tick infection, or during transmission stages (e.g. [1–10]). Further investigations have identified a number of factors that are involved with *B*. *burgdorferi* gene and protein regulation, including DNA-binding proteins, two-component sensory mechanisms, and enzymes that synthesize/degrade modified nucleotide alarmones (e.g. [1,11–18]). Detailed understanding of the regulatory networks at play during *B*. *burgdorferi* infection processes is essential to develop novel therapies that specifically target essential borrelial processes.

Transcriptomic investigations of spirochetal regulatory mechanisms during mammal or tick infection are complicated by the facts that *B*. *burgdorferi* do not achieve high concentrations during mammalian infection, and individual ticks do not contain substantial numbers of bacteria [1]. As proxies, researchers have profiled gene expression in cultured bacteria to model transcriptomic changes (e.g. [2,13,17,19–34]). Studies have been performed to compare *B*. *burgdorferi* transcriptomes in wild-type bacteria grown under different culture conditions, or a mutant versus its wild-type parent strain. While these have revealed important biological insights, they have been limited in several key ways. First, nearly all of these studies used microarrays to profile gene expression, and examined only annotated coding regions of the *B*. *burgdorferi* genome. More recently, RNA sequencing (RNA-Seq) has been used to profile *B*. *burgdorferi* transcriptomes, but analyses of those data were still limited to annotated ORFs [17,31,33], and thus ignored intergenic RNAs. Furthermore, most could not differentiate between sense (i.e. protein-encoding) and antisense transcripts. It is increasingly apparent that diverse bacteria produce numerous noncoding RNAs (ncRNAs) that regulate myriad biological processes [35,36]. Some ncRNAs hybridize with mRNAs to alter translation or stability, some regulate other cellular functions (e.g. 6S RNA), while others combine with proteins to form nucleoprotein complexes (e.g. the secretory signal recognition particle, SRP) [37–40]. To date, little is known about the existence of ncRNAs in *B*. *burgdorferi*. One study used homologies with other bacterial species to predict borrelial ncRNAs, confirmed production of 12 ncRNAs, and concluded that *B*. *burgdorferi* appears to contain only a few noncoding RNA molecules [41]. The intervening 12 years have shed very little new light on borrelial ncRNAs: a positive regulator of *rpoS* mRNA translation, *dsrA*, has been identified and characterized [14,42,43], and another locus was predicted to encode 6S RNA, but its transcription was not confirmed [37]. Both of those ncRNAs were identified in the present studies.

Another limitation of previously-published studies of the *B*. *burgdorferi* transcriptome is that, with the exception of a single study examining the transition from exponential growth into a starvation state [33], they were limited to a single time point and thus failed to define transcriptional dynamics as cultures progressed. Several examples are known of *B*. *burgdorferi* proteins being differentially expressed as cultures age [20,44,45]. Thus, it is possible that some previously-reported differences in mRNA expression levels may have been due to variations in culture ages, rather than the mutations or other conditions being investigated.

To address those questions and concerns, we performed RNAtag-Seq on multiple, independent *B*. *burgdorferi* cultures. Each culture was sampled at three different stages: early exponential, mid-exponential, and early stationary phases. RNAtag-seq is a novel method which allows for the multiplexing of up to 32 directional cDNA library preparations in a single reaction [46]. This method establishes stranded-ness by ligating DNA adapters directly to the 3’ ends of fragmented RNA, then using those tag sequences as priming sites for reverse transcription. A second adaptor is then ligated to the 3’ ends of the first strand cDNAs, followed by PCR amplification and Illumina sequencing. The combination of genomic DNA depletion, ligation of adapters directly to RNA, and specific (non-random priming) PCR enrichment generates a strand-identified library with very low levels of background [46]. The resulting data include reads derived from both sense and antisense strands of protein coding genes and from intergenic regions. The particularly low levels of background reads facilitated the detection of ncRNAs and mapping of transcript boundaries. The latter further enabled experimental assessment of algorithms that in silico predict Rho-independent termination sites.

## Materials and Methods

### Bacteria and culture conditions

*B*. *burgdorferi* B31-A3 is an infectious, clonal derivative of the species type strain B31, and contains all of the naturally-occurring DNA elements of the sequenced culture of strain B31 except cp9 [47–49]. Absence of cp9 does not have any detectable effects on *B*. *burgdorferi* physiology either during infection processes or in culture [50,51]. Immediately prior to these studies, the DNA content of B31-A3 cultures were assessed by multiplex PCR [52], to assess presence of all naturally-occurring plasmids.

*B*. *burgdorferi* were cultured in Barbour-Stoenner-Kelly II (BSK-II) liquid medium, prepared in-house [53]. Bacteria from glycerol stocks that had been frozen at -80°C were diluted 1:100 into 5 ml of fresh medium, then incubated at 35°C. Transfer of *B*. *burgdorferi* from -80°C to warmer growth conditions induces substantial changes in transcript and protein levels [54]. To prevent those effects from impacting upon our results, the initial 35°C cultures were grown until cell densities reached mid- exponential phase (1x10^7^ bacteria/ml), then diluted 1:100 into 30 ml of fresh BSK-II (to a final density of 1x10^5^ bacteria/ml), and again incubated at 35°C. Aliquots of those cultures were removed when bacterial densities reached early-exponential (1x10^6^ bacteria/ml), mid-exponential (1x10^7^ bacteria/ml), and stationary phases of growth (1 day after plateauing at 1x10^8^ bacteria/ml). For early-exponential cultures, 20 ml were required to reliably isolate sufficient RNA for library construction. For mid-exponential and stationary phase cultures, 5 ml was sufficient. Bacteria were harvested by centrifugation at 8200xG for 30 min at 4°C. Supernatants were decanted and the cell pellets were immediately resuspended in 1 ml of pre-warmed (60°C) TRIzol (Thermo-Fisher). These suspensions were stored at -80°C until processed.

### RNA isolation, library preparation, and RNA-Seq

Cell-TRIzol suspensions were thawed at room temperature. RNA was isolated from 500 μl of cell suspension using the Zymo RNA Direct-Zol miniprep kit (Zymo). Isolated RNA was eluted in 35 μl RNase-free water and stored at -80C. RNA quantity and integrity was assayed by microfluidic analysis using an Agilent 2100 Bioanalyzer and with the Agilent RNA 6000 Nano chip kit (Agilent) and only samples with a RIN score > 9 were used for library construction.

Illumina cDNA libraries were generated using the RNAtag-seq protocol as described [46]. Briefly, 1 μg of total RNA was fragmented, depleted of genomic DNA, dephosphorylated, then ligated to DNA adapters carrying 5’-AN8-3’ barcodes with a 5’ phosphate and a 3’ blocking group. Barcoded RNAs were pooled and depleted of rRNA using the RiboZero rRNA depletion kit (Epicentre). These pools of barcoded RNAs were converted to Illumina cDNA libraries in 3 main steps: (i) reverse transcription of the RNA using a primer designed to the constant region of the barcoded adaptor; (ii) degradation of the RNA and ligation of a second adaptor to the single-stranded cDNA; (iii) PCR amplification using primers that target the constant regions of the 3’ and 5’ ligated adaptors and contain the full sequence of the Illumina sequencing adaptors. cDNA libraries were sequenced on four lanes of an Illumina Nextseq 500.

For the analyses of RNAtag-Seq data, reads from each sample in the pool were identified based on their associated barcode using custom scripts [55]. Up to 1 mismatch in the barcode was allowed, with the caveat that it did not enable assignment to more than one barcode. Barcode sequences were removed from reads, and the reads from each sample were aligned to the *B*. *burgdorferi* B31 genome sequence using BWA [47,48,56]. Differential expression analysis was conducted with raw reads counts per gene using DESeq [57].

Sequences have been deposited in the NCBI Short Read Archive (SRA) under the Bioproject ID PRJNA339291 and Biosample IDs SAMN05587080, SAMN05589073, and SAMN05589074.

### Quantitative RT-PCR

Sets of bacterial cultures were grown in identical conditions described for those used for preparing RNA-Sequencing libraries. RNA was extracted and validated for quality as described above, with the addition of on-column DNase I digestion (Zymo). Isolated RNA was then converted to cDNA using the iScript cDNA synthesis kit (Bio-Rad). Quantitative RT-PCR (qRT-PCR) was performed essentially as previously described [58] using a Bio-Rad CFX96 cycler and oligonucleotide primers listed in S1 Table. Comparisons were made using the ΔCt method, using *ftsK* as a reference gene. As described below, *ftsK* was determined by RNA-Seq to be constitutively expressed under all culture conditions, whereas other transcripts that have previously been used as internal controls (*flaB* and *recA*) were observed to vary during cultivation.

### RNA sequencing data analyses: Identification of Rho-independent terminators and 5’ ends of transcripts

To identify potential Rho-independent terminators, in silico analyses of the *B*. *burgdorferi* B31 genome sequence were performed, then those results were compared with RNA-Seq data. The genome sequence was queried using three separate intrinsic terminator prediction programs: RNAMotif [59], TransTerm [60], and FindTerm [61], as implemented in the SIPHT pipeline [62]. If a potential terminator was identified any of these programs, it was included for further analysis. Each of these predictions were then manually annotated with an indicator of their relative location: the 3’ designation indicates putative Rho-independent terminators residing within 100bp of the stop codon or when read coverage supported continuous transcription to the terminator; the 5’ designation indicates putative Rho-independent terminators located within 150bp upstream or downstream of a start codon, but not meeting the criteria for 3’ designation; the Internal designation are putative Rho-independent terminators which are internal to coding sequences that do not meet the criteria for 3’ or 5’ designations; and those labeled IG are within intergenic regions but do not meet any of the other criteria. In some cases, genes which were very closely clustered would have terminators that met both the criteria for 5’ and 3’ annotations. These were annotated as 3’, consistent with a typical terminator ending transcription at the 3’ end of a gene. The inclusion of “A” in any of the annotations indicates that it is antisense to the direction of the gene with which it is associated. RNS-Seq read coverage surrounding each putative Rho-independent terminator sequence was then examined manually by several members of the research team.

To facilitate the detection of 5’ ends and ncRNAs, a custom script was utilized that determines putative transcriptional units. Coverage per position was averaged over a window of 10nt for each strand, then scanned 5’ to 3’ for positions with more coverage than the position before it. These positions are set as possible transcript boundaries (PTBs). The average coverage in windows between PTBs was calculated. Consecutive windows whose average coverage differed by less than 2.5 fold were joined, since the PTBs that separate them are more likely to represent localized dips in coverage than true transcript boundaries. The position with highest coverage in windows between pairs of consecutive PTBs was calculated. Coverage on either side of this position between the 5’ and 3’ adjacent PTBs was scanned, searching for a position with > 10-fold lower coverage. If such a position was not encountered, the PTB 5’ to the window was removed. This was done to filter out low “hills” of coverage that are unlikely to represent real, separate transcription units. Windows between remaining PTBs represent putative transcription units (PTUs). Short (<200 nt) PTUs that were directly adjacent (i.e. within one position) to long PTUs were joined together, from experience that such reads often correspond to 5’ and 3’ UTRs. The total and normalized (FPKM) abundance was calculated for each PTU. Each PTU was also annotated for its genomic location relative to other genes, to facilitate efficient differentiation among various classes of PTU. Similarly, the distance of the 5’ and 3’ ends of the PTUs from the 5’ and 3’ end, respectively, of the nearest ORF was calculated and a histogram of these distances was generated.

The 5’ ends of PTUs compared to the start codon were generated for each biological replicate at each time point. As 5’ ends can vary as a result of differential promoter usage in different conditions, 5’ ends were merged across replicates but not across time points. Several criteria were utilized when merging to further refine and increase the accuracy of 5’ end mapping: each must (1) reside 5’ of the start codon of its respective gene, (2) lie within 500bp of the start codon of its respective gene, (3) and finally when merging across biological replicates most distal identified 5’ end to its respective start codon was chosen. This last criterion was chosen because it gave the most reliable 5’ ends when compared to previously determined start sites.

### RNA sequencing data analyses: Noncoding RNAs

To facilitate detection of ncRNAs regardless of condition, read coverage histograms were merged across replicates and time points. This single file was then analyzed by determining PTUs as described above, but filtered by those which either did not overlap previously determined ORFs or overlapped them on opposite strands. These identified ncRNAs were further filtered to include only those putative ncRNAs that had a sum total of >5000 FPKM aligning to them across all 9 samples, to generate a single list of high confidence predicted transcripts. When identifying putative ncRNAs from the native plasmids, alterations were made to this cut-off, since relatively low expression from certain plasmids artificially inflated the FPKM-based expression of these loci. Effectively, both at the quantification level and visual level, the signal to noise ratio between background and expressed genes was reduced compared to the chromosome. To circumvent this, thresholds were determined by visual inspection of the read coverage plots for each particular genetic element to identify an appropriate cut-off, wherein ncRNAs expressed above that level were readily identified by visual inspection, while those below that level were not easily discerned above background.

## Results

### RNA-Sequencing of cultured *Borrelia burgdorferi*

To profile the global transcriptome of *B*. *burgdorferi*, we isolated RNA from triplicate cultures of wild-type strain B31-A3 at early-exponential and mid-exponential phases, and one day after reaching stationary phase. The assayed cultures were confirmed to contain all parental plasmids, using both multiplex PCR prior to RNA extraction and post hoc inspection of read mapping [52]. Total RNA was converted to Illumina cDNA libraries using the RNAtag-Seq protocol [55], and libraries were sequenced across four lanes on the Illumina NextSeq platform.

For each sample, at least 2 million reads were obtained, with an average of 17.9 million reads per sample. Targeted depletion of ribosomal RNA (rRNA) resulted in the vast majority of reads aligning to the sense and antisense strands of annotated ORFs, or to intergenic regions, with only ~4% of reads aligning to rRNA. Due to the modified bases in tRNAs, which impede the reverse transcription step in RNAtag-Seq, tRNAs are under-represented in the results [63,64].

There are 1386 annotated genes in the main chromosome and small DNA replicons of *B*. *burgdorferi* B31-A3 [47,48]. We detected expression of >10 fragments per kilobase per million reads aligning to annotated ORF (FPKMO) in at least one replicate for 92% of these annotated ORFs (Table 1 and S2 Table). Individual samples showed detectable expression of between 81.8% and 87.8% of the annotated genes, with an average of 84.9% of genes expressed at levels at or above 10 FPKMO. Pearson correlation between normalized gene expression of biological replicates exceeded 0.96 in most cases (S1 Fig).

**Table 1:** Sequencing metrics. Sequencing metrics for each sample including sample name, total reads, total reads aligned to the B31-MI genome, percentage of reads aligned to the B31-MI genome, and the percentage of reads mapping to ribosomal RNA loci.

Sample	Total Reads	Total Reads Aligned	Percent Reads Aligned	Percent Ribosomal
**A3 E1**	6.07E+06	2.41E+06	72.81	4.78
**A3 E2**	1.17E+07	5.09E+06	78.89	2.76
**A3 E3**	1.53E+07	6.79E+06	82.42	3.52
**A3 M1**	2.57E+06	8.65E+05	56.60	1.44
**A3 M2**	4.65E+07	1.31E+07	47.56	0.97
**A3 M3**	3.56E+07	1.10E+07	53.62	2.41
**A3 S1**	1.72E+07	2.62E+06	24.47	6.41
**A3 S2**	1.57E+07	3.31E+06	35.05	8.27
**A3 S3**	1.05E+07	2.91E+06	47.32	2.59

ORFs that did not produce significant levels of transcripts (i.e., consistently yielded less than 10 FPKMO) are listed in S3 Table. All but one of these ORFs are located on the small replicons/plasmids. They include genes of the resident cp32 prophages, and a large number of hypothetical plasmid-encoded proteins [65–67]

The RNA-Seq datasets were analyzed to identify differentially expressed genes, rho-independent terminators, 5’ ends of transcripts, and noncoding RNAs. Examples of each are described below and the results of all these analyses are provided in supplemental tables, allowing other researchers to examine data pertinent to their genes of interest. A caveat to this and every other study of cultured *B*. *burgdorferi* is that BSK-II medium is an artificial environment, in which some mammal-specific and tick-specific genes are transcribed simultaneously. It is possible, therefore, that some alternative transcripts are produced during the natural infectious cycle.

### *B*. *burgdorferi* differentially expresses numerous transcripts during cultivation

It is well known that *B*. *burgdorferi* and other bacteria change their transcriptional profiles as cultures mature [44,45,68]. To gain insight into changes in gene expression elicited by changes in growth phase, RNAtag-Seq libraries were generated from 3 independent cultures of *B*. *burgdorferi* B31-A3 grown in the same batch of medium and in the same 35°C incubator and harvested at the same three culture densities.

**Fig 1:**
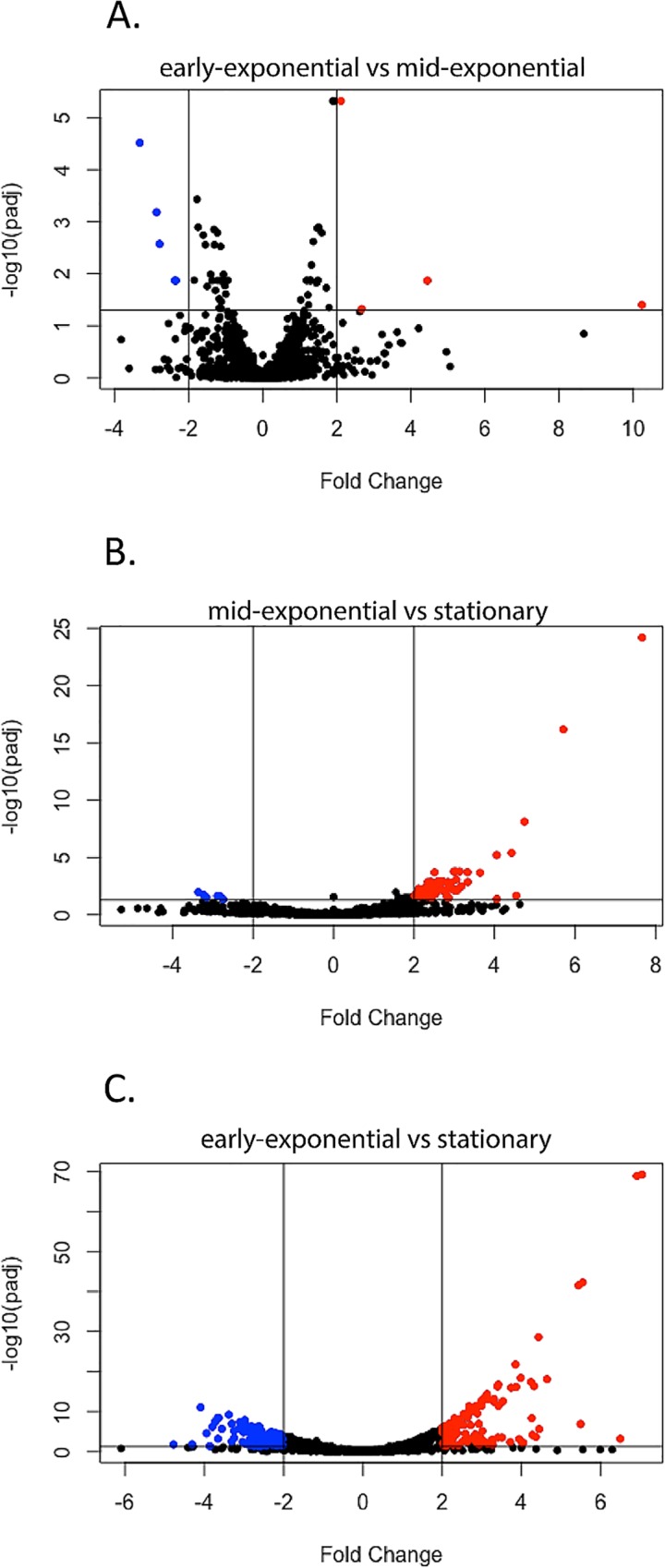
Volcano plots of differentially expressed genes. Fold changes between genes were plotted compared to adjusted p-value when comparing (A) early-exponential against mid-exponential, (B) mid-exponential against stationary, and (C) early-exponential against stationary. Criteria of >2X change in expression and <0.05 adjusted p-value were used to define significantly changed genes, and are shown on the plot with the appropriate limiting lines. Genes which met the criteria and were expressed at higher levels in a particular comparison are shown in red and those which were expressed at lower levels are shown in blue.

Two important observations were made that impact qRT-PCR analysis in this and other studies. First, transcripts that have been used in previous studies as “invariant” internal controls were seen to vary in expression levels during cultivation. Second, transcript levels of a substantial number of *B*. *burgdorferi* mRNAs changed as cultures progressed from early exponential into early stationary phase. A total of 243 of the annotated ORFs (17.6%) were found to be significantly differentially expressed (log_2_ fold change > 1X and a false-discovery rate < 0.05) among the different growth phases (Fig 1 and S4, S5, and S6 Tables).

**Fig 2:**
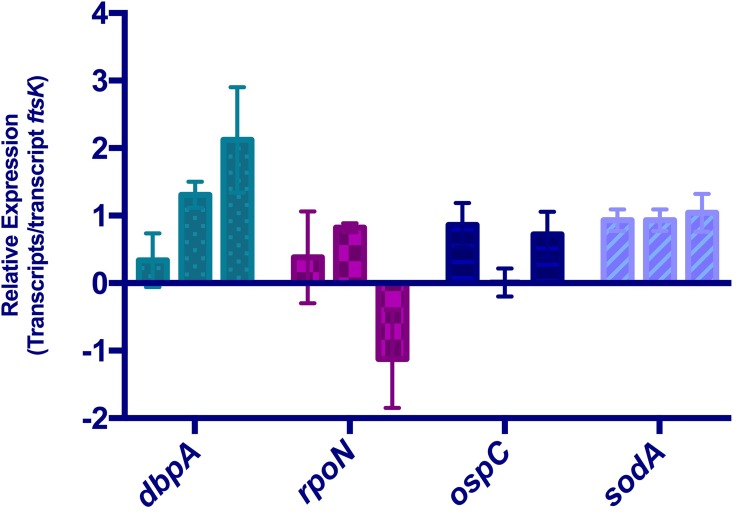
Quantitative RT-PCR analyses of select transcripts. Results for each transcript were compared with results for *ftsK* transcript of that culture. For each gene tested, bars from left to right are relative abundance during early-exponential, mid-exponential, and stationary phases of growth.

The *flaB* transcript is frequently used as a qRT-PCR reference transcript [58,69,70]. The *recA* mRNA has also been used on occasion as a reference, under the same assumption of invariability [16,18]. However, our RNA-Seq analyses consistently revealed differences in both *flaB* and *recA* mRNA levels when comparing early or mid-exponential to stationary phases (S4 and S5 Tables). For this reason, we mined our data for a more stably-expressed mRNA. The *ftsK* message was identified as being nearly unchanged across growth phases, varying at most by 0.98x. Therefore, all qRT-PCR results were analyzed using *ftsK* as the internal, constant standard (Fig 2).

RNA-Seq revealed 9 significant differences in annotated genes when comparing early-exponential and mid-exponential cultures. Four transcripts increased and 5 decreased during transition to mid-exponential stage. Of the four transcripts that increased, three are of unknown function, and the fourth is a putative bacteriophage integrase on small replicon lp56 (S4A Table). Of the five genes that were significantly reduced, four are *bpaB* genes of the cp32 prophages (S4B Table). Previous studies found that *bpaB* transcript levels decreased as *B*. *burgdorferi* growth rate increased [54].

In contrast, increased culture density to 10^8^ bacteria/ml was accompanied by significant changes in many transcripts. When comparing mid-exponential phase with stationary phase, 66 genes increased and 7 genes decreased in transcript abundance (S5A and S5B Table). When comparing early-exponential to stationary phase, 129 genes were expressed at higher levels and 98 genes were expressed at lower levels (S6A and S6B Table).

Transcripts of several genes important for infection and host surface adhesion were elevated during stationary phase, including *dbpA* and *dbpB* (2.4X and 4.5X, respectively). Among the genes that decreased in abundance during the exponential-stationary transition were factors involved in DNA repair and replication (*dnaA*, *polA*, *recD*, and *recB*), and genes involved in central metabolism (*pfs* and *metK*). Those observations are consistent with an overall slowdown of cellular metabolism and cessation of DNA replication.

Relative expression levels of select genes were also assessed by use of qRT-PCR, permitting comparisons of transcript level determination by the RNA-Seq and qRT-PCR methods. Transcripts that RNA-Seq indicated to increase, decrease, or remain stable across all three culture stages were assayed. When comparing early-exponential to stationary phase of growth, *dbpA* was significantly increased in transcript abundance and *rpoN* was significantly decreased in abundance, detected by both DEseq analysis of RNA-Seq and by qRT-PCR (Fig 2). When assayed by qRT-PCR and DESeq, *sodA* did not significantly change during cultivation. DESeq analysis of *ospC* indicated a dip in transcript abundance in mid-exponential phase, which was not detected by qRT-PCR. This disparity in detection by the two methods is being investigated further.

### *B*. *burgdorferi* contains an extensive untranscribed DNA sequence

**Fig 3:**
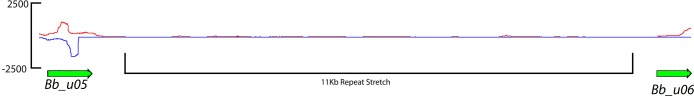
Example of an extensive, untranscribed sequence of *B*. *burgdorferi*. Read coverage histograms of the 11kb direct-repeat sequence within the naturally-occurring plasmid lp21. Red lines indicate relative transcript abundance from the + strand (left to right) and blue indicate relative transcript abundance from the–strand (right to left), and reside above (+) and below (-) the central axis. Genes are noted below coverage plots and directionality is indicated by arrows at the ends of genes. Relative orientation of genes on the X axis is consistent with RefSeq annotations. Coverage per base is given on the Y axis to the right of the plot.

*B*. *burgdorferi* strain B31 naturally carries a ca. 21kb linear replicon named lp21, which contains an 11kb stretch of 61-63bp tandem repeats [48]. Other strains of *B*. *burgdorferi* carry this same sequence attached to an end of the linear main chromosome [71]. The function of this extensive stretch of directly-repeated sequences has yet to be determined. Small numbers of copies of the repeated sequence occur on other native plasmids: approximately one and a half copies reside on lp28-3, partially overlapping ORF *Bb_H06*, and partial copies exist on lp28-4 and lp36, both of which are immediately 3’ of annotated ORFs. All three of those ORFs were transcribed in culture (S2 Table). While we detected abundant expression of lp21 operons that flank the 11kb repeat element, almost no reads mapped within the repeat stretch (Fig 3), suggesting that this region is not transcribed. The small number of dispersed reads that were computationally mapped within the repeat element are likely due to inappropriate mapping of reads from the lp28-3, lp28-4, and/or lp36 sequences. A caveat of mapping algorithms is that repeated sequences cannot be discriminated, so if a read aligns equally well to multiple loci, it will be randomly assigned to one of those loci. The absence of detectable transcription from the borrelial 11kb direct repeat sequence adds further to the mystery of why this unusual sequence is so well-conserved among *B*. *burgdorferi* strains.

### Mapping of transcript 5’ ends

Methods have been developed that can enrich for RNAs which carry a 5’ triphosphate and therefore may represent primary transcripts [72]. These allow for the differential examination of RNA-Seq (dRNA-Seq) libraries which are enriched in primary transcripts compared to those that are not. While such methods can facilitate identification of transcript start sites, data from RNAtag-Seq can also be analyzed to identify probable transcription starts. Newly-developed methods for analysis of RNA-Seq data have mapped start sites that agree well with both dRNA-Seq and methods such as 5’ RACE [73]. RNA-Seq was recently used by another group to identify the putative transcriptional promoters of the *B*. *burgdorferi tamB* and *bamA* genes [74]. To this end, we mined our RNAtag-Seq data for large increases in read coverage within a narrow window. Approximately 600–800 putative transcript 5’ ends in each of culture/condition dataset. A large proportion of these putative 5’ ends mapped within 40bp upstream of the nearest annotated start codon. All results are listed in S7 Table, to assist researchers in further investigating genes of interest.

The transcriptional start sites of several *B*. *burgdorferi* operons have previously been mapped by methods such as primer extension or 5’ rapid amplification of cDNA ends (5’ RACE) and more recently, RNA-Seq. The majority of previously-determined 5’ ends which were identified in our data set matched either exactly or within a few bases of our identified 5’ ends [74–79], adding to confidence that newly-identified 5’ ends are likely to be accurate start sites (Table 2).

**Table 2:** Comparison of predicted transcriptional start sites with previously identified transcriptional start sites. Comparison of genes with previously identified transcriptional start sites that were also identified by RNA-Seq. Columns list the gene, the previously-mapped start-site location, citation for that determination, and RNA-Seq determined sites during early-exponential, mid-exponential, and stationary phases of growth. For some operons, RNA-Seq algorithms identified different start sites from different cultures; in which case, all called start sites are listed.

Gene	Mapped 5’ End	Citation	RNA-Seq Early-exponential	RNA-Seq Mid-exponential	RNA-Seq Stationary
*ospC*	-20	[75]	-19/-21	-21	-16
*secA-sod*	-100	[77]	-100	-100	-100/-27
*bpuR*	-42	[15]	-44	-44	-44
*chbC*	-42	[78]	-24/-25/-28	-29/-41/-46/	-26/-24
*lon-1*	-19	[76]	Not detected	-17	-17
*tamB*	-13	[74]	Not Detected	-13	-13
*bamA*	-343	[74]	Not Detected	-348	-348

### Identification of intrinsic termination sites

Rho independent, or intrinsic, terminators can abruptly end extension of a transcript, or serve as regulatory sequences [80]. Prior to performing RNA-Seq, the *B*. *burgdorferi* B31 genome sequence was analyzed by combined use of three separate intrinsic terminator prediction programs. Those in silico analyses predicted the existence of 201 Rho-independent termination sites (S8 Table).

RNA-Seq data were then visually inspected for abrupt drops in mapped reads surrounding those sites, as expected from Rho-independent termination. Many of the predicted terminators were followed by distinct reductions reads mapped, although some exceptions were found. Some of these discrepancies may reflect the fact that Rho-independent terminator prediction algorithms were trained and vetted on *E*. *coli*, whose nucleic acid composition is substantially different from that of *B*. *burgdorferi* (51% vs. 29% G+C, respectively) [47,48,81].

**Fig 4:**
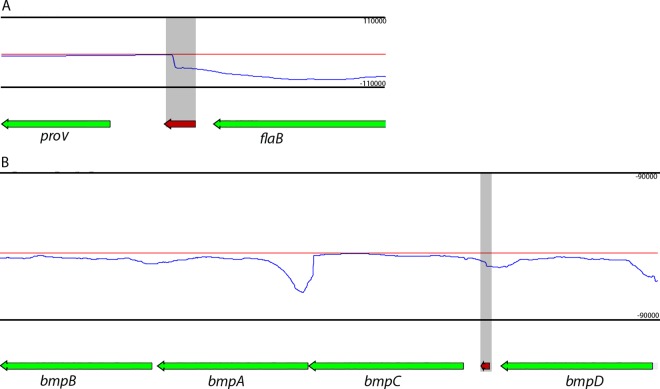
Examples of Rho-independent termination sites at the end of genes that were bioinformatically predicted and supported by transcriptomic data. **(A)** *flaB*; and **(B)** *bmpDCAB*. Thin red lines indicate transcript abundance from the + strand (left to right) and thin blue indicate transcript abundance from the–strand (right to left), and are shown above (+) and below (-) the central axis. Genes are noted below coverage plots and directionality is indicated by arrows at the ends of genes. Relative orientation of genes on the X axis is consistent with RefSeq annotations. Coverage per base is given on the Y axis to the left of the plot. Predicted Rho-independent terminators are indicated by red boxes on the same plane as the gene annotations and directionality is indicated by arrows. Terminators and the associated coverage at their location are highlighted by grey boxes.

Of the in silico predicted Rho-independent terminators that were supported by RNA-Seq data, 99 (49%) resided within 100bp 3’ of a stop codon, or had transcriptomic support for an extended 3’ UTR that ended at the predicted terminator. Examples include *flaB* and the *bmpDCAB* operon (Fig 4). The flagellin-encoding *flaB* gene is expressed at high levels ([82] and S2 Table), so a strong terminator would prevent read-through into the unrelated downstream gene. The *bmpDCAB* locus constitutes a complex operon. Two intrinsic terminators have previously been identified biochemically, residing between *bmpD* and *bmpC*, and between *bmpA* and *B* [83,84]. The computer algorithms predicted the *bmpD–bmpC* terminator, which was supported by RNA-Seq read mapping (Fig 4C). The *bmpA–bmpB* terminator was neither predicted nor apparent from experimental data. RNA-Seq read-mapping detected elevated levels of reads immediately 5’ of both *bmpD* and *bmpA*, consistent with the previously reported transcriptional start sites [84] (Fig 4C).

Intrinsic terminators residing within the 5’ UTR, or inside the coding region of an mRNA can have regulatory effects on transcriptional elongation and, therefore, protein expression [85]. Nineteen percent of the predicted terminators are located within 150bp of a start codon. The remaining percentage of identified intrinsic terminators resided either well within genes or were located intergenically yet not within 100bp of any annotated gene or putative ncRNA.

Other transcripts were associated with distinct decreases in coverage at their 3’ ends that did not overlap a predicted Rho-independent terminator, suggesting that these 3’ boundaries may be due to RNA processing of longer transcripts or that the sequences requirements for Rho-independent termination in *B*. *burgdorferi* vary significantly from those in other model organisms.

The 3’ ends of the majority of genes lacked any predicted intrinsic terminator and lacked discrete ends. These transcripts generally ended with regions of gradually declining transcript abundance after the stop codon. This gradual trailing off of transcript coverage could be due to a number of reasons, including Rho-dependent termination or degradation. Because of these uncertainties and the variability in length of decline, we did not comprehensively analyze 3’ ends of transcripts that did not contain apparent Rho-independent terminators.

### *B*. *burgdorfer*i evidently transcribes numerous noncoding RNAs

RNA-Seq has the unparalleled ability to investigate transcriptional activity from both strands of DNA. This allows the unbiased discovery of both intergenic noncoding RNAs (ncRNAs) and RNAs that are transcribed antisense to protein-coding ORFs (asRNA). To identify candidate *B*. *burgdorferi* ncRNAs, we mined our RNA-Seq data for relatively short (<425) putative transcription units located in non-coding regions of the genome or antisense to annotated protein encoding genes. This analysis yielded 351 putative ncRNAs (S9 Table), of which 129 were transcribed from the main linear chromosome, 82 from the resident cp32 prophages, and 140 from the remaining linear and circular plasmids. Slightly more than half (186) of the ncRNAs were transcribed antisense to annotated coding sequences. Sixty-two putative ncRNAs were encoded in intergenic regions without any overlap of known ORFs. One hundred three contained both antisense and intergenic sequences, of which 39 included sequences that are antisense to or overlap pseudogenes.

**Fig 5:**
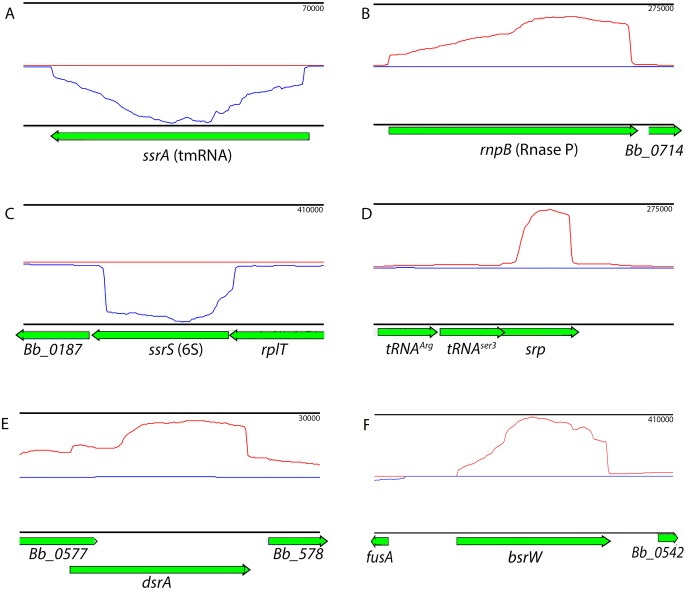
Examples of intergenic noncoding RNAs. Red lines indicate transcript abundance from the + strand (left to right) and blue indicate transcript abundance from the–strand (right to left), and are shown above (+) and below (-) the central axis. Genes are noted below coverage plots and directionality is given by arrows at the ends of genes. Relative orientation of genes on the X axis is consistent with RefSeq annotations. Coverage per base is given on the Y axis to the left of the plot. Neither *dsrA* (E) nor *6S* (C) are currently annotated in either GenBank or RFAM. *dsrA* is shown as the longest possible transcript described in [42].

Among the most highly expressed ncRNAs were homologs of stable regulatory and catalytic RNAs, including 6S, tmRNA, and the RNA subunits of RNase P and SRP (Fig 5). This study is the first to detect production of those ncRNAs in *B*. *burgdorferi*. Levels of the *srp* RNA were significantly increased upon entry into stationary phase. *dsrA*, the ncRNA that regulates translation of the RpoS alternative sigma factor [42], was also readily detected (Fig 5E).

One of the most highly-expressed transcripts under all culture conditions was a previously unannotated RNA of approximately 401 nucleotides. This novel RNA was designated *bsrW* (**b**orrelial **s**mall **R**NA W). It is encoded by a chromosomal locus that is between *fusA* and *Bb_0542* (Fig 5F). Although unannotated in the NCBI genome entry for *B*. *burgdorferi* B31, the sequence is conserved across much of the genus. Upon manual inspection, *bsrW* was found to contain a small ORF that is predicted to encode a 76 residue polypeptide. BLAST-P analyses (http://blast.ncbi.nlm.nih.gov/Blast.cgi) revealed homologies with other bacterial proteins that contain KTSC domains. Although such proteins are found in other bacterial species, their functions are not known. If *bsrW* does in fact encode a small ORF, it would have unusually long 5’ and 3’ untranslated regions (102 and 82 nucleotides, respectively).

**Fig 6:**
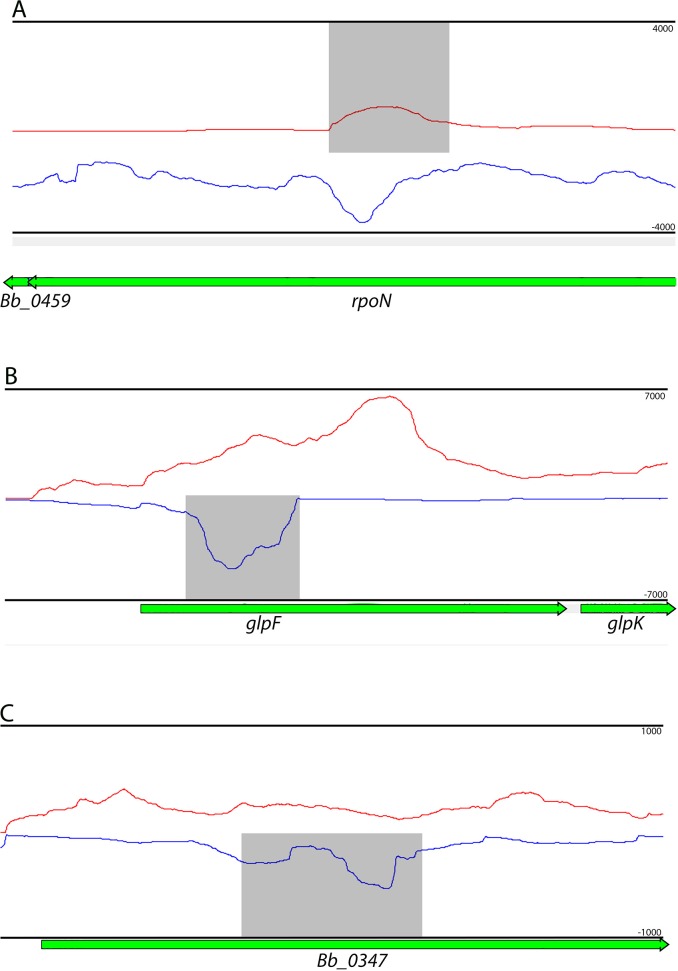
Examples of putative antisense noncoding RNAs. Read coverage histograms of previously antisense ncRNAs that overlap; *rpoN*, *glpF*, and ORF *Bb_0347*. Read Red lines indicate transcript abundance from the + strand (left to right) and blue indicate transcript abundance from the–strand (right to left), and are shown above (+) and below (-) the central axis. Genes are noted below coverage plots and directionality is given by arrows at the ends of genes. Relative orientation of genes on the X axis is consistent with RefSeq annotations. Coverage per base is given on the Y axis to the left of the plot. ncRNAs are highlighted by grey boxes determined by their predicted start and end sites determined in S8 Table. Identified Rho-independent terminators are indicated by red boxes, and directionality is shown by arrows at their ends.

These transcriptomic analyses also identified 186 transcripts that ran antisense to coding sequences. All were transcribed at levels above our conservative cut-off threshold, providing confidence that their production is of significance to *B*. *burgdorferi*. We did not further analyze these probable ncRNAs further, but provide the results as a service to investigators to explore targets of interest. Notably, many of these putative ncRNAs overlap genes important for infection and tick colonization, including *rpoN*, *glpF*, and *Bb_0347* (Fig 6) [86–88]. Antisense RNAs can have myriad effects on their cognate genes, including influencing transcriptional efficiency via polymerase competition or base pairing with complementary transcripts to prevent or alter transcript stability.

## Discussion

Transcriptomics revolutionized the study of biological systems in the early 2000s with the advent of the microarray. For the first time, researchers were able to investigate gene expression on a genome-wide scale in a single experiment. The field was once again revolutionized in 2009 with the development of RNA-Seq, utilizing the next-generation sequencing platforms. As the various technologies have matured, RNA-Seq has become the gold standard for transcriptomic investigations. The most recent iterations of RNA-Seq allow for the global comparison of gene expression patterns, and the unbiased identification of transcriptional start sites, transcriptional end sites, and previously undefined transcripts. To date, only a handful of studies have used RNA-Seq to explore the transcriptomes of *B*. *burgdorferi* or any other spirochete, and those focused almost exclusively on mapping transcripts from ORFs [17,31,33,89]. The present study represents the first detailed analysis of coding and non-coding transcripts in *B*. *burgdorferi* and the first to examine transcriptome variation and stability during culture aging.

Approximately 18% of annotated *B*. *burgdorferi* ORFs were found to be differentially expressed as cultures progressed, during standard cultivation conditions. In addition, as consequences of long-acting regulatory mechanisms that control *B*. *burgdorferi* gene expression, previous conditions encountered by a culture can have significant impacts on gene expression. For example, *B*. *burgdorferi* passaged from -80°C to 23°C exhibit significantly different transcript and protein patterns than do those that are passaged from 35°C to 23°C [54]. Those results indicate the importance of using identically parallel cultures when attempting to compare *B*. *burgdorferi* strains.

*B*. *burgdorferi* also appears to express a substantial number of noncoding RNAs, both from intergenic regions and antisense to coding sequences. Thus, as has been shown for many other bacteria, there is a strong probability that the Lyme disease spirochete utilizes ncRNAs to control transcriptional and post-transcriptional processes. Functional evaluation of these ncRNAs certainly merits further study.

Analyses of these RNA-Seq data gave insight on borrelial transcriptome architecture, including apparent transcriptional start and stop sites. In silico prediction algorithms were fairly effective at identifying Rho-independent terminator sequences, but were not perfect. The algorithms are generally based on studies of *E*. *coli*, which contains 51% G+C. Results of our studies can be used to refine those prediction programs for better analyses of low G+C bacteria such as *B*. *burgdorferi*. Evaluation of differences in read 5’ ends enabled identification of several previously-defined transcriptional start sites. Thus, many of the 5’ ends listed in S7 Table probably correspond with transcriptional start sites. Those data are presented to guide investigators in further studies of genes of interest, to be confirmed by additional methods such as primer extension or 5’-RACE. We note that those methods can yield incomplete pictures, especially when an operon utilizes more than one promoter/transcriptional start site. Combinations of RNA-Seq and another, independent method may provide the most accurate results.

Taken together, our findings provide key new insights into the transcriptional changes underlying *B*. *burgdorferi* progression through different growth phases during cultivation. Additionally, they offer a significantly more detailed map of the *B*. *burgdorferi* transcriptome that, along with the raw RNA-Seq data generated in this study, will help advance efforts to better understand this important pathogen.

## Supporting Information

S1 Fig Correlation between replicates. Log_2_ relative expression (FPKMO) comparing each replicate were plotted and Pearson’s correlations were calculated.
(EPS)

S1 Table PCR oligonucleotide primers used in these studies. (XLSX)

S2 Table Relative gene expression of annotated genes. The fragments per Kilobase per million fragments of ORF (FPKMO) were determined for each annotated gene. Column 1 contains the ORF identification number, and columns 2–10 contain the FPKMO lists for each of the 3 replicates of each of the 3 samples.
(XLSX)

S3 Table Annotated ORFs that were not readily transcribed in any culture. S2 Table, which containing the FPKMO expression levels of all 1386 annotated genes in the *B*. *burgdorferi* B31-A3 genome, was filtered to identify ORFs which were not present at greater than 10 FPKMO in any sample at any growth phase. Columns list each genetic element, ORF identification number, and expression levels in three biological replicates at all three phases of growth.
(XLSX)

S4 Table Transcripts with significantly different abundance in mid-exponential compared to early-exponential. **(A)** Genes are listed which were identified by DEseq as differentially expressed by meeting the criteria of >2X greater abundance and an adjusted p-value of <0.05. Four genes were identified which met these criteria. **(B)** Genes are listed which were identified by DEseq as differentially expressed by meeting the criteria of >2X lower abundance and an adjusted p-value of <0.05. Five genes were identified which met these criteria. In order from left to right columns list genetic element on which a given gene resides, the gene ID, the mean expression, the early-exponential expression, mid-exponential expression, fold change, log2 fold change, p-value, adjusted p-value, mid-exponential variance, and early-exponential variance.
(XLSX)

S5 Table Transcripts with significantly different abundance in stationary phase compared to mid-exponential. **(A)** Genes are listed which were identified by DEseq as differentially expressed by meeting the criteria of >2X greater abundance and an adjusted p-value of <0.05. Sixty-five genes were identified which met these criteria. **(B)** Genes are listed which were identified by DEseq as differentially expressed by meeting the criteria of >2X lower abundance and an adjusted p-value of <0.05. Seven genes were identified which met these criteria. In order from left to right columns list genetic element on which a given gene resides, the gene ID, the mean expression, the mid-exponential expression, stationary expression, fold change, log2 fold change, p-value, adjusted p-value, stationary variance, and mid-exponential variance.
(XLSX)

S6 Table Transcripts with significantly differen abundance in stationary phase compared to early-exponential. **(A)** Genes are listed which were identified by DEseq as differentially expressed by meeting the criteria of >2X greater abundance and an adjusted p-value of <0.05. One hundred and twenty-nine genes were identified which met these criteria. **(B)** Genes are listed which were identified by DEseq as differentially expressed by meeting the criteria of >2X lower abundance and an adjusted p-value of <0.05. Ninety-eight genes were identified which met these criteria. In order from left to right columns list genetic element on which a given gene resides, the gene ID, the mean expression, the early-exponential expression, stationary expression, fold change, log2 fold change, p-value, adjusted p-value, stationary variance, and early-exponential variance.
(XLSX)

S7 Table Putative transcript 5’ ends. **(A)** early-exponential phase cultures, **(B)** mid-exponential phase, and **(C)** stationary phase cultures. Listed putative 5’ ends include the ORF’s genetic element, putative start location, strand, distance from ORF start codon, and description of the ORF.
(XLSX)

S8 Table Bioinformatically predicted intrinsic termination sites. The full list of predicted intrinsic terminators. Included is are the genetic element, start location, strand, length, program predicted (T: TransTerm, R: RNAMotif, F: FindTerm, B: RNAMotif and TransTerm, X: RNAMotif and FindTerm, Z: RNAMotif, FindTerm, and TransTerm), score as described in materials and methods, and relative location compared to nearby genes.
(XLSX)

S9 Table Identified non-coding RNAs. Non-coding RNAs were identified as described in the materials and methods. Included are the genetic element, start position, end position, strand, relative location (I: Intergenic, A: Antisense, P: Pseudogene), length, associated genes, and those genes functions. Orders of numeric position and annotations are given according to order of the (+) strand. Note that Position 1 and Position 2 reflect start and stop sites differently depending on strand. For ncRNAs located on the (+) strand position 1 is the start and position 2 is the stop. For entries located on the (-) strand, position 1 is the end site and position 2 is the start site. Commas between associated genes and functional annotations indicate that the ncRNA overlaps both contiguously and “/” between them indicate that it is located intergenically between the two genes.
(XLSX)
